# Distinguishing Patients with Parkinson's Disease Subtypes from Normal Controls Based on Functional Network Regional Efficiencies

**DOI:** 10.1371/journal.pone.0115131

**Published:** 2014-12-22

**Authors:** Delong Zhang, Xian Liu, Jun Chen, Bo Liu

**Affiliations:** 1 Department of Radiology, Guangdong Provincial Hospital of Chinese Medicine, Guangzhou, China; 2 Guangzhou University of Chinese Medicine postdoctoral mobile research station, Guangzhou, China; Michigan State University, United States of America

## Abstract

Many studies have demonstrated that the pathophysiology and clinical symptoms of Parkinson's disease (PD) are inhomogeneous. However, the symptom-specific intrinsic neural activities underlying the PD subtypes are still not well understood. Here, 15 tremor-dominant PD patients, 10 non-tremor-dominant PD patients, and 20 matched normal controls (NCs) were recruited and underwent resting-state functional magnetic resonance imaging (fMRI). Functional brain networks were constructed based on randomly generated anatomical templates with and without the cerebellum. The regional network efficiencies (i.e., the local and global efficiencies) were further measured and used to distinguish subgroups of PD patients (i.e., with tremor-dominant PD and non-tremor-dominant PD) from the NCs using linear discriminant analysis. The results demonstrate that the subtype-specific functional networks were small-world-organized and that the network regional efficiency could discriminate among the individual PD subgroups and the NCs. Brain regions involved in distinguishing between the study groups included the basal ganglia (i.e., the caudate and putamen), limbic regions (i.e., the hippocampus and thalamus), the cerebellum, and other cerebral regions (e.g., the insula, cingulum, and calcarine sulcus). In particular, the performances of the regional local efficiency in the functional network were better than those of the global efficiency, and the performances of global efficiency were dependent on the inclusion of the cerebellum in the analysis. These findings provide new evidence for the neurological basis of differences between PD subtypes and suggest that the cerebellum may play different roles in the pathologies of different PD subtypes. The present study demonstrated the power of the combination of graph-based network analysis and discrimination analysis in elucidating the neural basis of different PD subtypes.

## Introduction

Parkinson's disease (PD) is a movement-related neurodegenerative disorder that has a broad range of clinical symptoms, including resting tremor, slowness of movements, rigidity, and gait disturbance [1]. The resting tremor (i.e., T-subtype) and akinesia/rigidity (i.e., AR-subtype) [2], in particular, have been demonstrated to be the two predominant subtypes of PD. Although the neuropathological findings associated with these PD subtypes have been shown to differ [3], [4], the neural substrate underlying these subtypes is still largely unknown.

The pathological changes observed in tremor-dominant PD patients differ from those observed in non-tremor-dominant PD [5]. PD is traditionally attributed to a neurodegenerative process in the dopaminergic nigrostriatal system (e.g., the basal ganglia) [6]. However, striatal dopamine depletion and the consequent dysfunction of the basal ganglia are of more relevance to akinesia/rigidity than to resting tremors [7]. Accordingly, the akinesia/rigidity subtype, rather than the tremor-dominant subtype, is typically responsive to dopamine treatment. In fact, several studies have indicated that nigrostriatal dopaminergic loss itself might be insufficient to result in PD tremor [5], [8] and thus that other neural systems (e.g., the cerebellum) may be involved in the generation of PD tremor [9]. In particular, cumulative evidence suggests that the cerebellum may play a substantial role in generating the clinical symptoms of PD [10]. Alterations in neural activity in the cerebellum may be induced not only by the pathological changes observed in PD but may also reflect compensatory effects in PD patients [10]. In particular, neural compensatory activity in the cerebellum has been observed to be an important component of the neural mechanism of tremor generation in PD. Neuroimaging studies have suggested that striatal dopamine depletion in PD cause damage to broadly distributed neural circuits [11] (e.g., the cortico–striatal–thalamic [6], [12] and cerebello-thalamo-cortical circuits) [13]. These neural activity circuits may have different roles in generating the distinct clinical symptoms of PD. For instance, the cortico–striatal–thalamic circuit may be involved in generating akinesia/rigidity [6]; however, the integration of the cortico-striatal-thalamic and the cerebello-thalamo-cortical circuits may be critical for tremor generation in PD. More importantly, several studies have shown that the neural activities of the basal ganglia and of the cerebellum are highly associated with each other [10] and that these circuits may be a component of the larger PD-related brain network. Thus, researchers have gradually realized that a network (e.g., connectome) perspective may be more appropriate for understanding the neural basis of PD, and several studies have demonstrated that functional brain networks are disrupted in PD patients [14]–[16]. The findings of these studies suggest that PD-related neural activity could be sensitively reflected in both the local and global levels of the functional brain network.

In the present study, we investigated the brain functional network topology related to two PD patient subtypes (i.e., tremor-dominant PD and non-tremor-dominant PD) via combining graph-based network analysis and linear discriminant analysis. We also explored the role of the cerebellum in the PD functional network. To this end, we collected resting-state functional MRI (R-fMRI) data from 15 tremor-dominant PD patients, 10 non-tremor-dominant PD patients, and 20 matched normal controls (NCs). Two types of functional brain networks for each participant were constructed based on high-resolution, randomly generated anatomical templates with and without the cerebellum. A graph-based network efficiency metric was then employed to characterize the topological organization of each functional brain network. Then, a non-parameter permutation test and a linear discriminant analysis were combined to determine whether the regional network efficiency could distinguish the mixed PD patients and the PD subtypes from the NCs.

## Materials and Methods

### Participants

Twenty-five right-handed PD patients and 20 matched NCs were recruited for the present study. The PD patients underwent neurological examinations and were scored using the Unified Parkinson's Disease Rating Scale (UPDRS), Hoehn & Yahr Scale (H-Y stage), and Mini-Mental State Examination (MMSE). All the patients were diagnosed according to the UK PD Brain Bank Criteria. A total of 15 tremor-dominant PD patients with classical parkinsonian resting tremor with (n = 13) or without (n = 2) action or postural tremor and 10 non-tremor-dominant patients with bradykinesia (n = 5) or akinesia (n = 5) were collected. Patients in advanced stages of PD (H-Y > = 4) or with cognitive impairments (MMSE <28), secondary parkinsonism, atypical parkinsonian disease or a history of any substance dependence, head trauma, or claustrophobia were excluded from the study. In the hours before and during MRI scanning, the patients were not given any medication, to avoid measuring the effects of the medications on patients as much as possible. Table 1 lists clinical and demographic information about the study participants.

**Table 1:** Demographic information for and clinical characteristics of the participants.

	NC (*n = *20)	TPD (*n = *15)	NTPD (*n* = 10)	*p-*Value
Gender	11 M/9 F	8 M/7 F	6 M/4 F	0.90^a^
Age (yrs)	42–78 (59.2±8.7)	37–81 (60.5±11.8)	46–78 (63.1±10.11)	0.37^b^
Edu (yrs)	0–22 (11.4 ± 5.0)	0–20 (9.8±4.2)	0–20 (7.8±3.2)	0.14^b^
ID (yrs)	-	0.42–6 (2.5±1.7)	1–7 (2.6±2.06)	-
MMSE	-	29.0–30 (29.8±0.05)	29.1–31 (30±0.07)	-
UPDRS	-	4–49 (27.3±14.3)	9–74 (31.8±21.2)	-
H-Y	-	1–3 (2.25±0.91)	1–3 (2.2±0.9)	-

Data are presented as minimum - maximum (mean ± SD). PD, Parkinson's disease; NC, normal control; TPD, Tremor-dominant PD; NTPD, Non-tremor-dominant PD; MMSE, Mini-Mental State Examination; UPDRS, Unified Parkinson's Disease Rating Scale; H-Y, Hoehn & Yahr Scale; Edu, education in years; ID, illness duration

a The *p*-value was obtained using a two-tailed Pearson chi-squared test.

b The *p*-values were obtained using two-sample, two-tail t tests.

All participants gave written, informed consent before participating in the present study. This study was approved by the Institutional Review Board of the Guangzhou University of Traditional Chinese Medicine.

### Image Acquisition

All participants were scanned using a 1.5T Siemens Avanto MR scanner (Siemens Magnetom Avanto, Erlangen, Germany) with a 12-channel phased-array head coil in the Department of Radiology of the Second Affiliated Hospital of Guangzhou University of Traditional Chinese Medicine. During data acquisition, the participants were asked to lie quietly in the MR scanner with their eyes closed. Resting-state functional images were acquired using a gradient-echo echo-planar imaging (GE-EPI) sequence. R-fMRI images were collected using an echo-planar imaging sequence with the following parameters: 30 axial slices; repetition time  = 2000 ms; echo time  = 39 ms; gap  = 1 mm; slice thickness  = 4 mm; flip angle  = 90°; matrix  = 64×64; field of view  = 240×240 mm^2^, and 180 volumes. The 3D structural images were acquired using a T1-weighted MP-RAGE sequence: TR  = 1160 ms, TE  = 4.21 ms, TI  = 900 ms, flip angle  = 15°, FOV  = 256×256 mm^2^, matrix  = 512×512, slice thickness  = 1 mm, and 192 sagittal slices.

### Data Preprocessing

R-fMRI data preprocessing was performed using the GRETNA toolbox (http://www.nitrc.org/projects/gretna/) and SPM8 software (http://www.fil.ion.ucl.ac.uk/spm/software/spm8/). For each participant, the first 5 volumes were discarded to allow for scanner stabilization. The remaining R-fMRI data were then corrected for the intra-volume acquisition time delay between slices and geometrical displacements due to head movement. After checking the head motion parameters, none of the participants were excluded based on the criterion of a displacement of >3 mm or an angular rotation of >3° in any direction. Notably, the head-motion profiles (i.e., the summary scalars of both gross and micro head motion) were matched between the PD patients and NCs (for all, *p*>0.10). All realigned functional data were spatially normalized to Montreal Neurological Institute (MNI) space using an optimal 12-parameter affine transformation and nonlinear deformations. The normalized R-fMRI data were then resampled to a 3-mm isotropic resolution and smoothed using a 4-mm isotropic kernel. The resulting images were temporal bandpass filtered (0.01–0.1 Hz), and linear trends were removed. Finally, several nuisance signals, including 24-parameter head-motion profiles [17], mean white matter (WM) and cerebrospinal fluid (CSF), were removed from each voxel's time course via regression analysis. The global signal was not removed using regression analysis [18]–[20] due to controversies surrounding the removal of global signals during preprocessing [21], [22].

### Network construction

**Figure 1:**
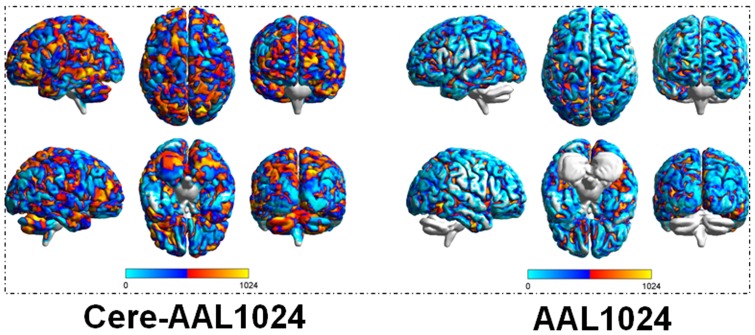
Templates used in the present study. a, the template with 1024 regions containing the cerebellum; b, the template with 1024 regions without cerebellum.

To uncover the global organization of regions throughout the whole brain, we constructed functional weighted networks for individual brain regions. The networks were constructed with the nodes corresponding to brain regions and the edges to interregional functional connectivity (FC). The network nodes were defined using a high-resolution, randomly generated anatomical atlas [23]. In the present study, two automated anatomical labeling (AAL) templates, constructed using the AAL atlas with and without the cerebellum, were independently applied to parcellate the cerebral cortex. Both atlases were parcellated into 1024 random anatomical regions of interest (ROI) to generate the anatomical templates (cere-AAL1024 and AAL1024; Fig. 1).

The Pearson correlations between the processed time series of any pair of ROIs were calculated, and the edges were defined as the FC strength between ROIs. These correlation coefficients were normalized using Fisher's r-to-z transformation, as follows: 
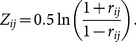
(1)


In parallel, two sets of functional networks (N×N, where N = 1024 is the number of ROIs) for each participant, corresponding to the two templates, were constructed. To further remove spurious interregional correlations, only those correlations whose corresponding *p*-values were below a threshold of statistical significance (*p*<0.05, Bonferroni-corrected) were retained [24] in the weighted functional networks. The analysis in the present study was restricted to examining positive connectivity due to the ambiguous interpretation of negative connections [21], [22].

### Network analysis

The constructed functional weighted networks were further fed into graph-based network efficiency analyses. Here, the global and local efficiencies were calculated to characterize parallel information flow within the networks of the PD patients (i.e., in both the tremor-dominant PD patients and the non-tremor-dominant PD patients) and the NCs.

The global network efficiency is a measure of a network's capacity for parallel information transfer between nodes via multiple series of edges [25]. Briefly, the global efficiency for a network 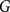 is defined as
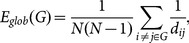
(2)where 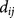 is the shortest path length between node 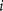 and node 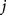 in 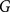 and is calculated as the smallest sum of edge lengths among all of the possible paths from node 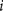 to node 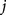.

The local network efficiency was calculated as the mean of the local efficiencies across all nodes within a network. The local efficiency of 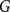 is defined as
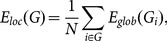
(3)where 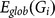 is the global efficiency of 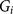, the sub-graph composed of the neighbors of node 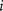.

The global and local efficiencies were normalized by the corresponding mean derived from 100 random networks. The topological organization of the weighted functional network was investigated using the normalized global efficiency (i.e., Lambda) and local efficiency (i.e., Gamma). Specifically, a network is thought to be a small-world network if it has a normalized local efficiency greater than 1 and a normalized global efficiency approximately equal to 1. The ratio of Gamma to Lambda (i.e., Sigma  =  Gamma/Lambda) was used to measure the small-world property of the network.

The nodal efficiency was measured to characterize the nodal properties of these functional networks. The nodal efficiency measures the mean shortest path length between a given node 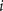 and all of the other nodes in the network. It quantifies the importance of node 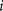
for information communication within a network, and is given as
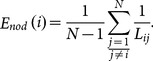
(4)


### Classification validation

To investigate whether the network nodal properties carry enough discriminative information to distinguish the PD patients from the NCs, we investigated whether the network nodal efficiency metrics (the global and local efficiencies) could be used to determine a pattern that performed well in distinguishing PD patients from NCs. Here, discrimination analysis was used to identify a distinguishing pattern that incorporated a feature selection approach.

Before the classification of subjects as PD patients or NCs was validated, feature selection was performed to reduce the dimensions of the data. For feature selection, significant between-group differences in network efficiency were inferred using nonparametric permutation tests [26], [27] (*p*<0.05, uncorrected). Briefly, for each network metric, we estimated the *t*-statistic to determine whether a between-group difference existed, and we then randomly assigned the parameter values into two groups to recalculate the *t-*statistic for the two randomized groups. We repeated this process for 10,000 permutations and obtained 10,000 *t*-statistics; we were thus able to estimate an empirical distribution of the group difference. The 95% confidence interval for the empirical distribution, generated using a two-tailed test, was used to determine the significance levels of the between-group differences. Before these permutation tests, multiple linear regressions were used to remove the effects of age and gender.

**Figure 2:**
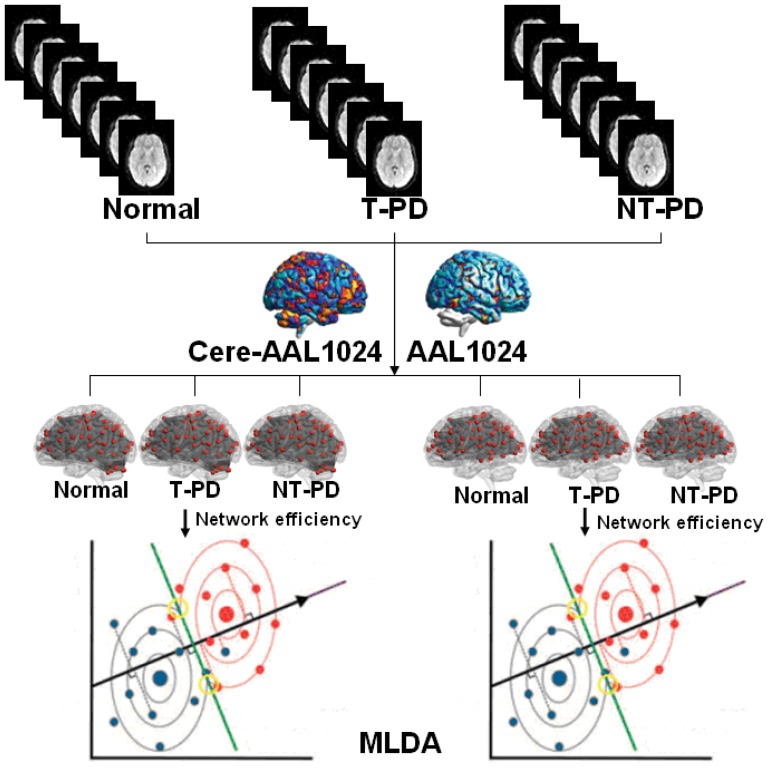
The data processing framework of the present study. Normal, normal controls; T-PD, tremor-dominant PD; NT-PD, non-tremor-dominant PD.

Maximum uncertainty linear discriminate analysis (MLDA) [28] classification was applied to classify the subjects as a PD patient with tremor-dominant or non-tremor-dominant PD or as a NC. Leave-one-out cross-validation (LOOCV) was used to validate the performance of the linear classifier. Specifically, using the LOOCV approach, the sample data were separated sequentially into the training data and the testing data; the training data were used to fit parameters of the classifier, and the testing data were used to test the performance of the classifier. The data processing framework of the present study is shown in Fig. 2.

### Validation analysis

The PD subtype classification findings obtained via analyses of network node efficiencies together with linear discrimination analysis were further validated using network property metrics (i.e., the clustering coefficient, 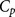, and the path length, 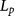). For a graph 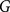 with 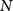 nodes and 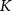 edges, 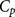
and 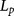 are calculated as
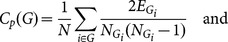
(5)

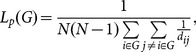
(6)where 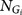 and 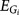 are the numbers of nodes and edges, respectively, within the subgraph 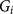, which is composed of neighbors of node 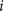, and 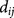 is the minimum number of edges required to travel from node 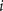 to 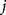.

## Results

### Network topological organization

**Figure 3:**
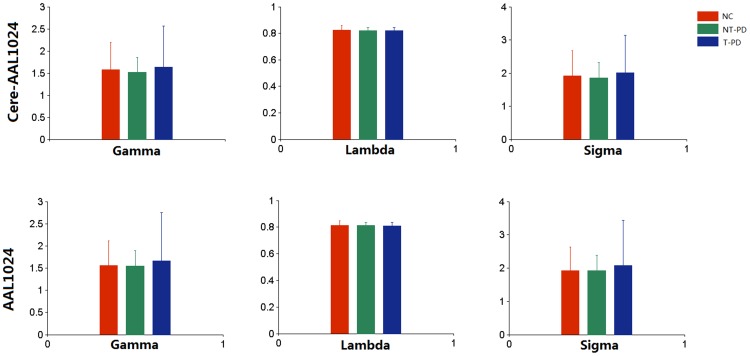
Spatial properties of the whole-brain functional networks. The network properties are depicted in terms of the network efficiency. NC, normal controls; T-PD, tremor-dominant PD; NT-PD, non-tremor-dominant PD.

We constructed two types of functional weighted networks, with and without the cerebellum, for the PD patients and the NCs, and the topological organizations of these functional networks were further analyzed using graph-based network analysis. All functional brain networks were found to be significantly small-world-organized in the NCs as well as in the PD patients, suggesting the presence of high-efficiency information translation in the functional networks of PD patients. The details are shown in Fig. 3.

### Distinguishing PD patients from normal individuals

The regional efficiencies (i.e., the local and global efficiencies associated with given regions) of the functional weighted networks were investigated. We found that differences in regional efficiencies of the functional weighted networks were able to classify the PD patients (i.e., both tremor-dominant and non-tremor PD patients) from the NCs. However, the regional local and global efficiencies in the functional networks performed differently in distinguishing the PD patients from the NCs. The regional local efficiency of the two types of functional networks both performed well in distinguishing the PD patients from the NCs. In contrast, the regional global efficiencies for the cere-AAL1024 and AAL1024 networks yielded inconsistent results. The global efficiency of the network corresponding to the cere-AAL1024 template rather than that of the AAL-1024 template could effectively distinguish the PD patients from the NCs, suggesting that the cerebellum may influence the global efficiency of the functional network in PD patients (Table 2).

**Table 2:** The performances of brain network parameters in distinguishing PD subtypes from NCs.

Performance	LE^a^	GE^a^	LE^b^	GE^b^
Sensitivity^1^	1.00	0.85	0.75	0.30
Specificity^1^	0.80	0.72	0.96	0.48
Accuracy^1^	0.89	0.77	0.86	0.40
Sensitivity^2^	0.95	0.85	0.80	0.75
Specificity^2^	1.00	0.93	0.86	0.86
Accuracy^2^	0.97	0.88	0.82	0.80
Sensitivity^3^	0.85	0.75	0.90	0.50
Specificity^3^	1.00	0.90	1.00	0.60
Accuracy^3^	0.90	0.80	0.93	0.53
Sensitivity^4^	0.87	0.86	0.87	1.00
Specificity^4^	1.00	1.00	1.00	1.00
Accuracy^4^	0.92	0.92	0.92	1.00

LE, local efficiency; GE, global efficiency;

a AAL1024 template including the cerebellum;

b AAL1024 template without cerebellum;

1 mixted PD - NCs;

2 tremor-dominant PD - NCs;

3 non-tremor-dominant PD - NCs;

4 tremor-dominant PD - non-tremor-dominant PD.

### Distinguishing PD subtypes from NCs

The regional efficiencies of the functional brain networks for patients with tremor-dominant and non-tremor-dominant PD, considered separately, were able to distinguish these patient groups from the NCs better than the regional efficiencies for the mixed group of PD patients. However, the regional efficiency of the functional network was not equally effective in distinguishing the tremor-dominant PD patients and the non-tremor-dominant PD patients from the NCs. The local efficiency of the functional brain networks was able to discriminate both the tremor-dominant and non-tremor-dominant PD patients from the NCs. Global efficiencies derived from the functional brain network related to the cere-AAL2014 template were better able to distinguish patients with different PD subtypes (i.e., tremor-dominant and non-tremor-dominant PD) from the NCs. However, the global efficiency of the functional brain network generated using the AAL1024 template distinguished tremor-dominant PD patients from NCs but failed to distinguish the non-tremor PD patients from the NCs. These results are shown in Table 2.

### Discriminating regions of the functional brain networks

In the present study, we investigated the role of feature selection in distinguishing the mixed group of PD patients from NCs. We found that the feature selection approach significantly influenced the performance of the classifier. The accuracy of the classifier performance without feature selection was almost at a random level (S1 Table).

**Figure 4:**
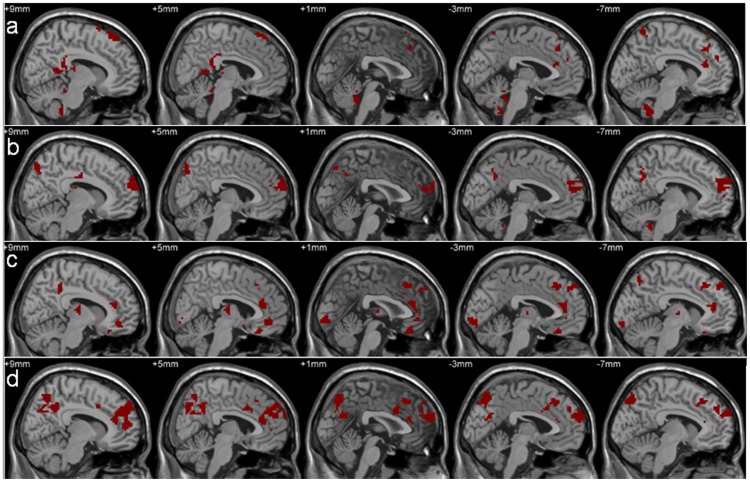
Regions capable of identifying the mixed PD patients from the NCs. a, the local efficiency related to cere-AAL1024; b, the global efficiency associated with cere-AAL1024; c, the local efficiency for AAL1024; d, the global efficiency for AAL1024.

By using a feature selection approach, we determined the regions that acquired distinguishing information that could be used to discriminate between the mixed group of PD patients and the NCs. The nodal efficiency of the functional brain network demonstrated the presence of significant network disruption in many regions in the mixed group of PD patients, and these regions associated with network disruptions were predominantly distributed across the limbic system, cerebellum, brainstem, basal ganglia, and the frontal, temporal and parietal cortex (Fig. 4).

**Figure 5:**
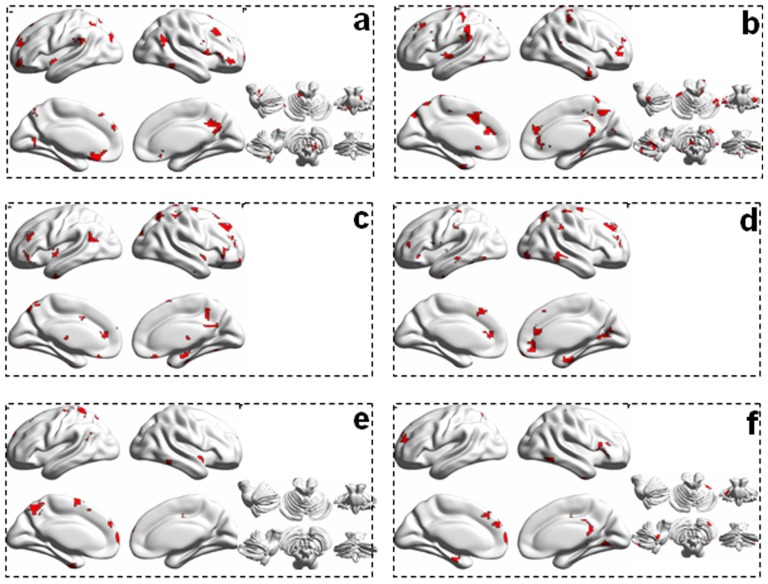
Distinguishing regions for distinguishing PD patients from the NCs. a-d are related to the local efficiency, and e-f is related to the global efficiency. a, c, and e correspod to the tremor-dominant PD classificantion compared with the NCs, and b, d, and f correspond to the classification of non-tremor-dominant PD compared with the NCs.

We further explored the discriminating regions that performed well in distinguishing the individual PD subtypes from the NCs. We found that the information required to distinguish the PD subtype from the NCs was predominantly obtained from observations of the limbic system (e.g., bilateral hippocampus and thalamus), basal ganglia (e.g., bilateral caudate and left putamen), cerebellum, insula and cingular cortex regions. These results are shown in Fig. 5.

### Distinguishing PD subtypes

The network nodal efficiency (i.e., the local and global efficiencies) was also used to distinguish between the PD patient subtypes and NCs. The results suggested that the network nodal efficiency could also effectively discriminate the different subtypes of PD from each other. The network nodal efficiency patterns derived from the networks generated using the cere-AAL1024 and the AAL1024 templates were both able to perform well in the classification of PD subtypes (Table 2). We further explored the regions for which network data for the two types of networks used were able to distinguish between groups of subjects. These regions, with variations in the local efficiency pattern, were predominantly distributed across the bilateral cerebellum, cortical regions such as the middle/superior temporal cortex, the precuneus, and the left postcentral gyrus. When examining global efficiency data across regions of the brain, the distinguishing regions included the right cerebellum, insula, and posterior cingular cortex, and the left fusiform, superior/middle temporal gyrus, and supplementary motor area.

### Validation of the findings

The findings of the present study were validated using the 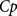
and 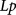 metrics. The brain networks of the PD patients and the NCs were all small-world organized (S1 Fig.). When study subjects were classified, we found that the 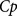
rather than the 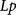
performed well in distinguishing PD patients from NCs. More importantly, these findings were robust for both networks generated using the cere-AAL1024 and AAL1024 templates. These results are shown in S2 Table.

## Discussion

In the present study, we measured the topological organization of the functional brain networks of PD patients and analyzed regional network efficiency to distinguish the mixed PD group and PD subtype groups from the NCs. The main findings demonstrated that analysis of the regional network efficiency (i.e., the local and global efficiencies) could effectively distinguish the tremor-dominant PD patients and the non-tremor-dominant PD patients from the NCs and that this ability to distinguish among the groups was influenced by the cerebellum.

The present study demonstrated that brain functional network efficiency could distinguish the PD patients from the NCs. PD is commonly attributed to dysfunction of basal ganglia circuits (i.e., cortico-striatal-thalamic loop), triggered by insufficient numbers of dopaminergic nigrostriatal neurons [6]. Because PD is a neurodegenerative disorder, even a focal lesion in PD may affect widely distributed neural systems [11], [29]. Autopsy studies have indicated that the pathophysiology of PD goes beyond neuronal degeneration within the nigrostriatal pathway and that pathological findings are found extensively throughout the brainstem, basal ganglia, and frontal and parietal cortex [30]. Thus, exploring brain activity from a network perspective is necessary to investigate the pathophysiology of PD [31]. Using graph theoretical measures, the spatial topological organization of the functional brain networks related to PD has been investigated previously in both local networks (e.g., the motor network [32]) and in whole-brain networks [15]. These studies suggest that the functional network in PD patients is altered on multiple levels (e.g., both the global and local levels). Given that network properties were significantly altered in the PD patients relative to the NCs, the network properties should convey information that can be used to distinguish the PD patients from the NCs. This hypothesis was directly verified in the present study; we found that alterations in network efficiency were able to discriminate the PD patients from the NCs.

The local and global efficiencies of the functional brain networks performed differently in differentiating the mixed PD group from the NCs. Specifically, we found that the local network efficiency, rather than the global efficiency, consistently performed well in identifying PD. The local efficiency is indicative of the ease of information transfer in the immediate neighborhood of each node within a network. Thus, the finding that local efficiency provides information that is useful for PD discrimination might suggest that obvious changes have occurred in connections between individual regions and adjacent regions in PD patients within functional brain networks. We also found that the performance of the global efficiency in identifying PD patients was dependent on the type of functional network that was examined (i.e., networks with or without the cerebellum). Further analysis showed that the global network efficiency performed differently in distinguishing between individual subtypes of PD. The network types influenced the performance of global efficiency data in distinguishing the non-tremor-dominant PD patients from the NCs. However, the ability to identify tremor-dominant PD was independent of the network type examined. These findings suggest that the physiological basis of tremor-dominant and non-tremor-dominant PD differ and that the cerebellum may play an important role in global network information translation.

Many studies have indicated that the patterns of dopaminergic neurodegeneration [33], the neural activity circuits [34], [35], and the response to dopaminergic treatment [36] observed in tremor-dominant PD patients are different from those observed in patients with akinetic-rigid symptoms. Accordingly, previous studies have shown that the neural substrates of tremor-dominant and non-tremor-dominant PD are quite different [7]. In the present study, we distinguished tremor-dominant and non-tremor-dominant PD patients, separately, from NCs. We found that local efficiency data still performed well in identifying tremor-dominant PD and non-tremor-dominant PD. However, the global efficiency of the network is not able to identify tremor-dominant PD and non-tremor-dominant PD equally well. Specifically, the global efficiency performed well in distinguishing the tremor-dominant PD patients from the NCs but the ability of the global efficiency to identify non-tremor-dominant PD was significantly dependent on the cerebellum. Thus, in functional networks that include the cerebellum, the global efficiency is able to distinguish the non-tremor-dominant PD patients from the NCs. These findings can also be used to understand why the global efficiency was not able to distinguish the mixed group of PD patients from the NCs. One important reason for this inability is that the effects of the global efficiency are restricted to the identification of non-tremor-dominant PD.

The cerebellum may play different roles in the functional networks observed in tremor-dominant and non-tremor-dominant PD. We found that the cerebellum significantly influenced network properties, which in turn influenced the ability to distinguish between non-tremor PD patients and NCs. Previous studies have shown that the cerebellum plays important roles in the pathology of PD [10]. The cerebellum is anatomically connected with other brain regions, such as the basal ganglia; such anatomical connections could provide a structural basis for the functional integration of regions linked to the cerebellum with other brain regions in PD patients [10]. For instance, a previous study showed that deep brain stimulation of the subthalamic nucleus was able to reduce blood flow in the cerebellum [37]. In the present study, we found that the cerebellum significantly influences the global efficiency of the brain network. Because the global efficiency is a measure of the capacity for parallel information transfer within a network [38], the cerebellum may play an important role in this process.

In the present study, certain regions, such as the basal ganglia, limbic regions (e.g., thalamus), cerebellum, and several cerebral regions (e.g., the insula, cingulum, and calcarine sulcus), were observed to be able to distinguish individual PD subtypes from the NCs. Previous studies have demonstrated that neural circuits (e.g., the cortico–striatal–thalamic and cerebello-thalamo-cortical circuits) play important roles in the pathophysiology of PD [7], [9]. As mentioned above, previous studies have shown that the cerebellum and basal ganglia are anatomically connected and form an integrated functional network of relevance to PD [10]. In this functional network, cortical regions such as the motor cortex may be critical for integrating these subcortical neural circuits [7]. In agreement with previous findings, the present study also showed that the neural activity in these regions in PD patients could be mapped onto the brain's intrinsic neural network and that it could be reflected in the network efficiency metric and examined further to improve the ability to discriminate between PD patients and NCs. It should be noted that the network efficiency (i.e., the global efficiency and local efficiency) and the harmonic metric (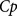 and 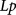) of the nodes of the brain network performed differently in their ability to identify PD subtypes, although these metrics could effectively reflect the network topology in the PD patients. We found that the harmonic metric was not sensitive to differences in nodal properties in the brain network between PD patients and the NCs in classification analyses. Our results suggest that the 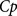 performed well in terms of both distinguishing the mixed PD patient group (subtype PD) from the NCs and classifying the PD subtypes, while the 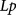 metric did not work well (S2 Table). These observations collectively suggest that the abilities of local variables (i.e., the local efficiency and 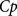), rather than global variables (i.e., the global efficiency and 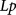) to translate information about the nodes in the functional network played important roles in distinguishing PD patients from NCs. In addition, we also found that the performance of the harmonic metric in distinguishing among the study participants was also not sensitive to the influence of the cerebellum on network topological properties. Thus, our findings demonstrate that network topology metrics played different roles in distinguishing PD patients from NCs. Compared with the harmonic metric, the network efficiency performed well in discriminating individual PD subtypes from NCs. Further, the present study directly demonstrated that the network nodal efficiency metric could also be effective at discriminate between PD subtypes. More importantly, we could obtain the network nodal efficiency pattern with power discrimination information for the networks related to both the cere-AAL1024 and the AAL1024 templates. These findings suggest that multiple regions besides the cerebellum had altered network efficiencies, which could form a pattern that could be used to effectively discriminate between different PD subtypes. These observations differed from the results obtained when attempting to distinguish mixed PD patients from the NCs. The pattern that was used to distinguish the mixed PD patients (subtype PD) from the NCs was characterized by the involvement of cortical regions (e.g., the insula, cingulum, and calcarine), the cerebellum and the subcortical regions (e.g., the basal gangli), while the pattern that included only cortical regions (e.g., the bilateral middle/superior temporal cortex and precuneus, and the left posterior central gyrus) was observed to distinguish patients with different PD subtypes from each other. Previous findings [7] suggest that neural activity in cortical regions, rather than pathological changes in subcortical regions, may play important roles in discriminating PD subtypes from each other. Overall, the present study may provide a new perspective with which to explore the neural basis of the pathology of individual PD subtypes.

Several issues should be addressed in future work. First, the sample size in this study was small, which may affect the conclusions of this study. Second, the present study revealed the regions in PD patients with altered regional efficiency; whether these regions could construct a sub-network should be further investigated. Third, although the network efficiency has been shown to effectively reflect the spatial topological organization of a network, other metrics of functional brain networks should also be used to identify PD subtypes in the framework of the present study.

## Conclusions

The present study measured the spatial organization of the functional brain network of PD patients and examined regional network efficiency to discriminate PD patients from NCs. We found that regional efficiency data carry enough information to identify PD patients; however, the local efficiency and the global efficiency performed differently. The local efficiency effectively distinguished the PD patients from the NCs; the global efficiency was able to distinguish the tremor-dominant PD patients from NCs but was dependent on the cerebellum to distinguish non-tremor-dominant PD patients from the NCs. The present study suggests that functional network efficiency is altered in PD patients and demonstrates the importance of the cerebellum in functional brain networks. This study also showed the different neural substrates of tremor-dominant and non-tremor-dominant PD.

## Supporting Information

S1 Fig
**Spatial properties of the whole-brain functional networks.** The network category is the same with that of Fig. 3, except for that the network properties are depicted in terms of the 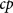
and 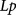.(TIF)

S1 Table **Classifier performances of the mixed PD from NCs without feature selection.** LE, local efficiency; GE, global efficiency; ^a^AAL1024 template with cerebellum; ^b^AAL1024 template without cerebellum.
(DOC)

S2 Table **Classifier performance based on the network nodal properties.** ^a^AAL1024 template with cerebellum; ^b^AAL1024 template without cerebellum; NC, normal control; T, tremor PD; NT, non-tremor PD; the content of the check indicates the sensitivity/specificity/accuracy of the classifier performance, respectively.
(DOC)
