# Supplementary figures and images for: Distinguishing Patients with Parkinson's Disease Subtypes from Normal Controls Based on Functional Network Regional Efficiencies
Source: PLoS One. 2014 Dec 22;9(12):e115131. doi: 10.1371/journal.pone.0115131 (PMC4274088; doi:10.1371/journal.pone.0115131)

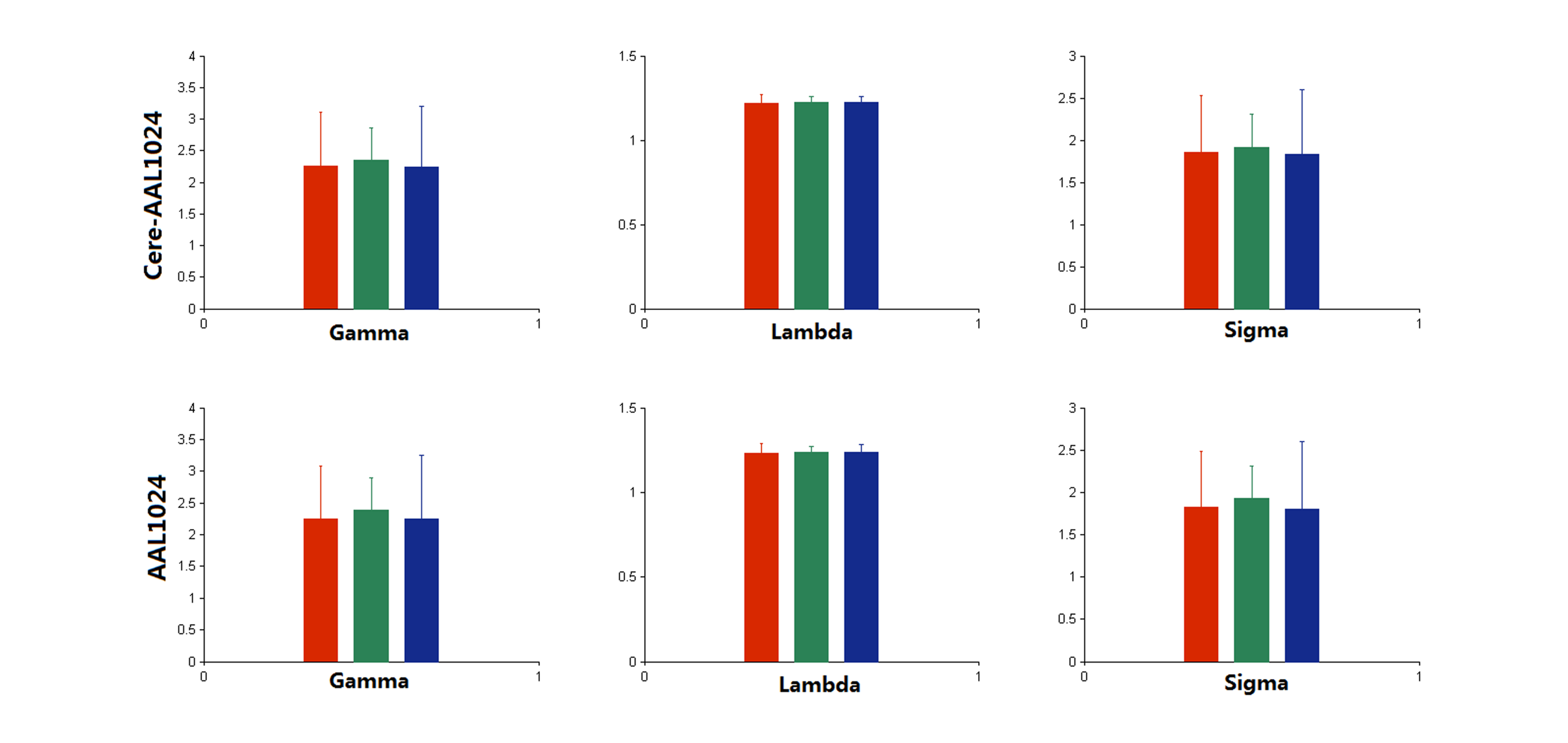

Supplement: S1 Fig — Spatial properties of the whole-brain functional networks. The network category is the same with that of Fig. 3, except for that the network properties are depicted in terms of the and . (TIF) [file pone.0115131.s001.tif]
