# Supplementary material for: Distinguishing Patients with Parkinson's Disease Subtypes from Normal Controls Based on Functional Network Regional Efficiencies
Source: PLoS One. 2014 Dec 22;9(12):e115131. doi: 10.1371/journal.pone.0115131 (PMC4274088; doi:10.1371/journal.pone.0115131)
Supplement: S1 Table — Classifier performances of the mixed PD from NCs without feature selection. LE, local efficiency; GE, global efficiency; aAAL1024 template with cerebellum; bAAL1024 template without cerebellum. (DOC) [file pone.0115131.s002.doc]

**Table S1.**

| **Performance** | **LE^a^** | **GE^a^** | **LE^b^** | **GE^b^** |
| --- | --- | --- | --- | --- |
| Sensitivity | 0.36 | 0.40 | 0.40 | 0.44 |
| Specificity | 0.25 | 0.35 | 0.30 | 0.40 |
| Accuracy | 0.31 | 0.37 | 0.35 | 0.42 |
