# Supplementary material for: Distinguishing Patients with Parkinson's Disease Subtypes from Normal Controls Based on Functional Network Regional Efficiencies
Source: PLoS One. 2014 Dec 22;9(12):e115131. doi: 10.1371/journal.pone.0115131 (PMC4274088; doi:10.1371/journal.pone.0115131)
Supplement: S2 Table — Classifier performance based on the network nodal properties. aAAL1024 template with cerebellum; bAAL1024 template without cerebellum; NC, normal control; T, tremor PD; NT, non-tremor PD; the content of the check indicates the sensitivity/specificity/accuracy of the classifier performance, respectively. (DOC) [file pone.0115131.s003.doc]

**Table S2.**

|  | **Cp^a^** | **Lp^a^** | **Cp^b^** | **Lp^b^** |
| --- | --- | --- | --- | --- |
| **NC-PD** | 1.00/1.00/1.00 | 0.60/0.65/0.62 | 0.96/1.00/0.98 | 0.68/0.50/0.60 |
| **NC-T** | 0.93/0.95/0.94 | 0.80/0.65/0.71 | 1.00/0.90/0.94 | 0.73/0.60/0.65 |
| **NC-NT** | 1.00/0.85/0.90 | 0.80/0.65/0.70 | 0.90/0.85/0.87 | 0.60/0.50/0.53 |
| **T-NT** | 1.00/0.86/0.92 | 0.80/0.60/0.68 | 1.00/0.93/0.96 | 0.80/0.80/0.80 |
